# PpCRN7 and PpCRN20 of *Phythophthora parasitica* regulate plant cell death leading to enhancement of host susceptibility

**DOI:** 10.1186/s12870-019-2129-8

**Published:** 2019-12-06

**Authors:** Heros J. Maximo, Ronaldo J. D. Dalio, Renata O. Dias, Celso G. Litholdo, Henrique L. Felizatti, Marcos A. Machado

**Affiliations:** 1 Biotechnology Laboratory, Centro de Citricultura Sylvio Moreira/Instituto Agronômico (IAC), Cordeirópolis, SP Brazil; 2 Instituto de Química, Universidade de São Paulo (USP), São Paulo, SP Brazil; 3 Instituto de Matemática, Física e Computação Científica, Universidade Estadual de Campinas (UNICAMP), Campinas, SP Brazil

**Keywords:** Crinkler effectors, Hemibiotrophic, Oomycetes, *Citrus*, Hypersensitivity response, Virulence

## Abstract

**Background:**

*Phytophthora* species secrete cytoplasmic effectors from a family named Crinkler (CRN), which are characterised by the presence of conserved specific domains in the N- and C-terminal regions. *P. parasitica* causes disease in a wide range of host plants, however the role of CRN effectors in these interactions remains unclear. Here, we aimed to: (i) identify candidate *CRN* encoding genes in *P. parasitica* genomes; (ii) evaluate the transcriptional expression of *PpCRN* (*Phytophthora parasitica* Crinkler candidate) during the *P. parasitica* interaction with *Citrus sunki* (high susceptible) and *Poncirus trifoliata* (resistant); and (iii) functionally characterize two *PpCRN*s in the model plant *Nicotiana benthamiana*.

**Results:**

Our in silico analyses identified 80 putative *PpCRN* effectors in the genome of *P. parasitica* isolate ‘IAC 01/95.1’. Transcriptional analysis revealed differential gene expression of 20 *PpCRN* candidates during the interaction with the susceptible *Citrus sunki* and the resistant *Poncirus trifoliata*. We have also found that *P. parasitica* is able to recognize different citrus hosts and accordingly modulates *PpCRN*s expression. Additionally, two PpCRN effectors, namely PpCRN7 and PpCRN20, were further characterized via transient gene expression in *N. benthamiana* leaves. The elicitin INF-1-induced Hypersensitivity Response (HR) was increased by an additive effect driven by *PpCRN7* expression, whereas *PpCRN20* expression suppressed HR response in *N. benthamiana* leaves. Despite contrasting functions related to HR, both effectors increased the susceptibility of plants to *P. parasitica*.

**Conclusions:**

PpCRN7 and PpCRN20 have the ability to increase *P. parasitica* pathogenicity and may play important roles at different stages of infection. These PpCRN-associated mechanisms are now targets of biotechnological studies aiming to break pathogen’s virulence and to promote plant resistance.

## Background

Plant-pathogen interactions are a ruthless battle, as the pathogens strive to invade host tissues in order to obtain nutrients and complete their life cycle. On the other hand, plants attempt to restrict the pathogen invasion and colonization to ensure its own survival. Frequently, the pathogen’s attack strategy relies on the secretion of effector proteins that functionally modulate the interaction with the host plant at early times of infection and colonization [1–3]. Plants recognize conserved pathogen-associated molecular patterns (PAMPs) and trigger the pattern-triggered immunity (PTI) for defense. To break this immunity process, pathogens secrete their repertoire of effectors, promoting the effector-triggered susceptibility (ETS) in the plant [4, 5].

*Phytophthora* is a genus of oomycete that forms a group of eukaryotic microorganisms classified within Stramenopiles, which are notable plant pathogens, affecting a wide variety of plants, and causing an extensive damage in natural and cultivated ecosystems [6]. The most notorious oomycete belong to genus *Phytophthora* (meaning “plant destroyer” in Greek) that includes more than 100 species, arguably the most devastating pathogens of dicot plants [7]. *Phytophthora spp*. are hemibiotrophic pathogens [8] and several species of *Phytophthora* have already been described as causal agents of disease in Citrus, including the most important and widespread *P. nicotiane* (= *P. parasitica*) Dastur and *P. citrophthora* (Sm. & Sm.) Leonian, [9]. However, that also includes *P. boehmeriae* Saw, *P. cactorum* (Lebert & Cohn) Srhöter, *P. capsici* Leonian, *P. cinnamomi* Rands, *P. ciricola* Saw, *P. drechsleri* Tucker, *P. hibernalis* Meat, *P. megasperma* Drechsler, *P. palmivora* (Butler) Butler, *P. nicotiane* (= *P. nicotiane* B. De Haan var.). *Phytophthora spp.* can cause several diseases in citrus depending on the plant tissue that is infected, with root rot and trunk gummosis [10, 11]. Citrus gummosis disease is considered one of the most serious diseases affecting citrus industry worldwide, causing significant economic losses in several regions [12].

Species belonging to the genus *Phytophthora* had their genome sequenced, revealing that this oomycete presents several putative effector protein-coding genes that can potentially manipulate the physiology of host plants. Such effectors can either promote virulence or activate the host defense system [13, 14]. Generally, effector proteins are classified, based on their location, as apoplastic (when secreted in the extracellular matrix) or cytoplasmic (when translocated into the host cells) [13, 15]. The cytoplasmic effectors, such as RxLR or Crinkler (CRN), are modular proteins that carry conserved domains in their N-terminal portion [2, 13, 16, 17]. These N-terminal conserved domains are related to the translocation of the effector to the host cytoplasm and define the effectors superfamily. At the C-terminal region, there are more diversified types of domains that are not related to protein translocation, but instead, to the specific functions of effectors [15].

The CRN proteins mostly share the N-terminal motifs LxLFLAK (leucine–any amino acid–phenylalanine–leucine–alanine–lysine) that is highly conserved [15]. The majority of the CRN effectors also carry a DWL domain and an HVLVXXP motif downstream the LxLFLAK motif [15]. These effector proteins are predominantly associated with necrosis induction; however; some of them may inhibit or suppress programmed cell death (PCD), triggered by PAMPs [18–20].

To investigate the role of *P. parasitica* PpCRN effector family during plant-pathogen interactions, this work presents: (i) identification of candidate *PpCRN* genes in *P. parasitica* isolate ‘IAC_01/95.1’ and genome comparison with genome data available of other isolates; (ii) transcriptional gene analysis of candidate *PpCRN*s expression during *P. parasitica* interaction with the susceptible *Citrus sunki* and the resistant *Poncirus trifoliata*; and (iii) functional characterization of two *PpCRN*s in *P. parasitica* interaction with the model plant *Nicotiana benthamiana*.

## Results

### Candidate Crinkler (CRN) effectors of *P. parasitica* (PpCRN)

Here, we explored the available genomes of *P. parasitica* deposited under the international project “*Phytophthora parasitica* genome initiative” database (https://www.ncbi.nlm.nih.gov/assembly/?term=phytophthora%20parasitica) to obtain the genome data of *P. parasitica* isolates from different hosts and geographic origins, to study the CRN effectors.

**Fig. 1:**
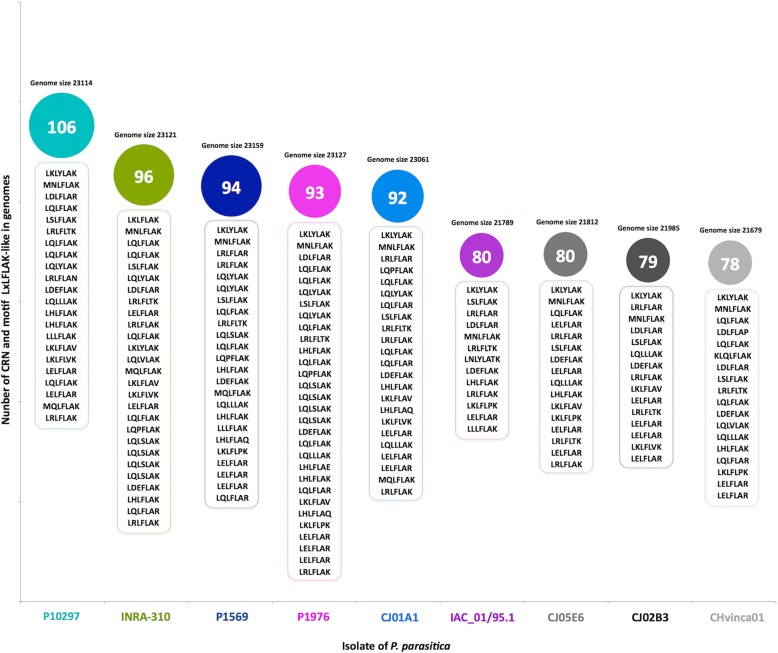
Prediction and identification of *Phytophthora parasitica* PpCRN effectors. Number of PpCRN predicted and found in each *P. parasitica* isolate genome, and the diversity of LxLFLAK motifs, in the isolates ‘IAC_01/95.1’, ‘P1569.1’, ‘P1976.1’, ‘INRA-310.3’, ‘P10297.1’, ‘CJ01A1.1’, ‘CJ05E6.1’, ‘CHvinca01.1’, ‘CJ02B3.1’

We identified 80 candidate genes encoding PpCRN effector proteins in the genome of *P. parasitica* isolate ‘IAC_01/95.1’ (Fig. 1). Similarly, candidate *PpCRN* effectors were found in the genomes of other *P. parasitica* isolates, with the isolate ‘P10297’ showing the highest number of *PpCRN* candidates (106), and the isolate ‘CHvinca01’ the least number (78). The conserved LxLFLAK motif was identified in several PpCRN candidates from distinct *P. parasitica* genomes, but it showed a variation in terms of quantity and sequence diversity from one genome to another (Fig. 1). Secretory signal peptides were predicted in only six PpCRN candidates, namely PpCRN2, PpCRN5, PpCRN7, PpCRN10, PpCRN14 and PpCRN20, which corresponds to 7.5% of total candidate proteins identified in the genome of isolate ‘IAC 01/95.1’.

**Fig. 2:**
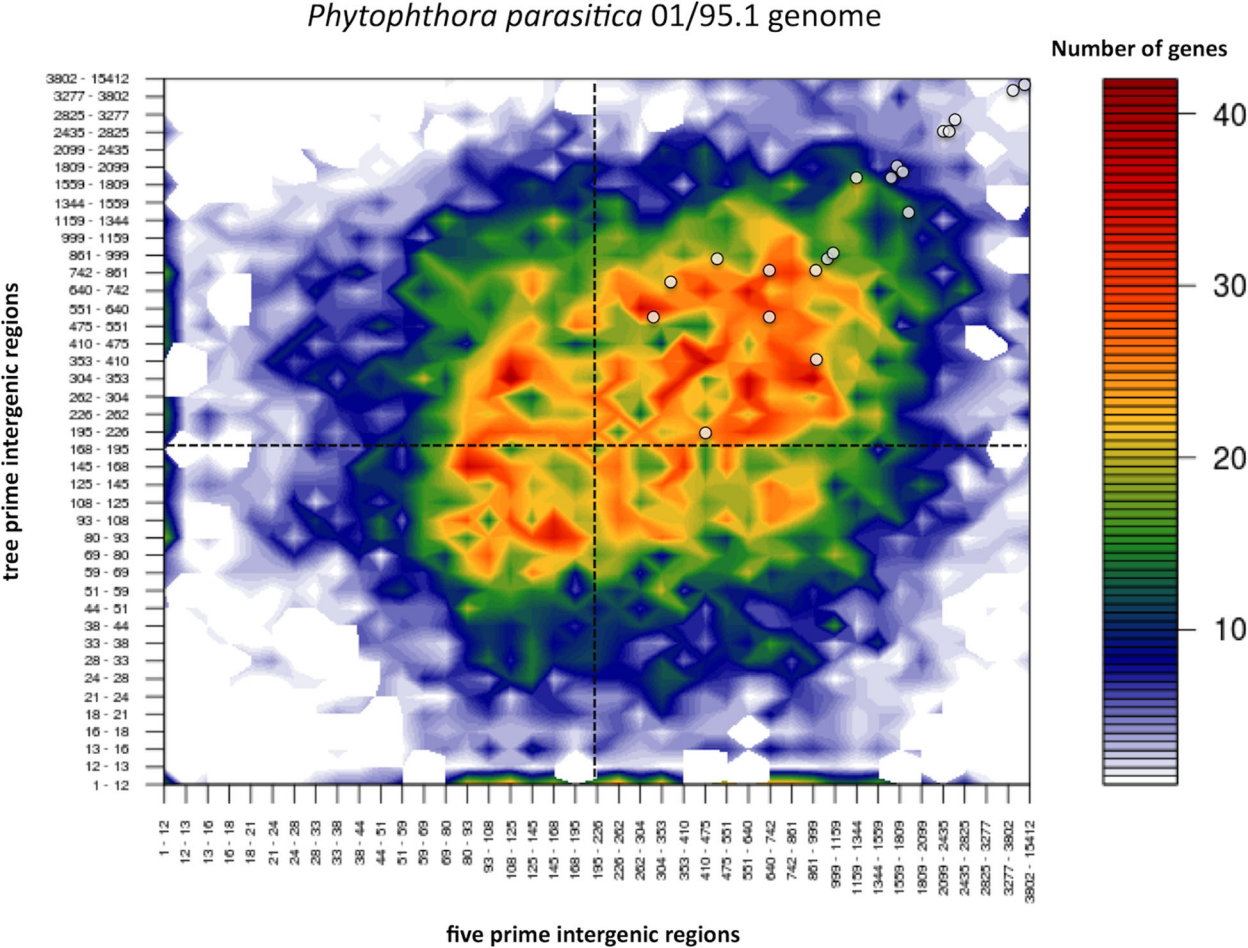
Genome architecture of *P. parasitica* isolate ‘IAC_01/95.1’ containing 20 candidate *PpCRN* effectors. All *PpCRNs* are localized at the sparse region of the genome. The heat map shows the number of genes at the same spot on the chart

The genome architecture can provide information about the function, regulation and adaptation of genes [15]. In *Phytophthora* species, some regions are rich in replicate and sparse genes, which are related to pathogenicity, including effector-coding genes. The *P. parasitica* genome shows a heterogeneous distribution according to the size of the intergenic regions. The genome architecture of the *P. parasitica* isolate IAC 01_95 is shown in Fig. 2. The flanking distance (intergenic region) between neighbouring genes provides a measurement of the local distribution of gene density, which can be plotted into two-dimensional graph based on the length of intergenic regions between neighbouring genes, at their 5′- and 3′-end. The genome architecture of *P. parasitica* shows that 20 selected *CRN* genes are located at the sparse region of the genome (Fig. 2). In the sparse region, due to its plasticity, the chances of emerging a new effector or simply evolving an already existing protein is more likely to happen than in the dense region.

**Fig. 3:**
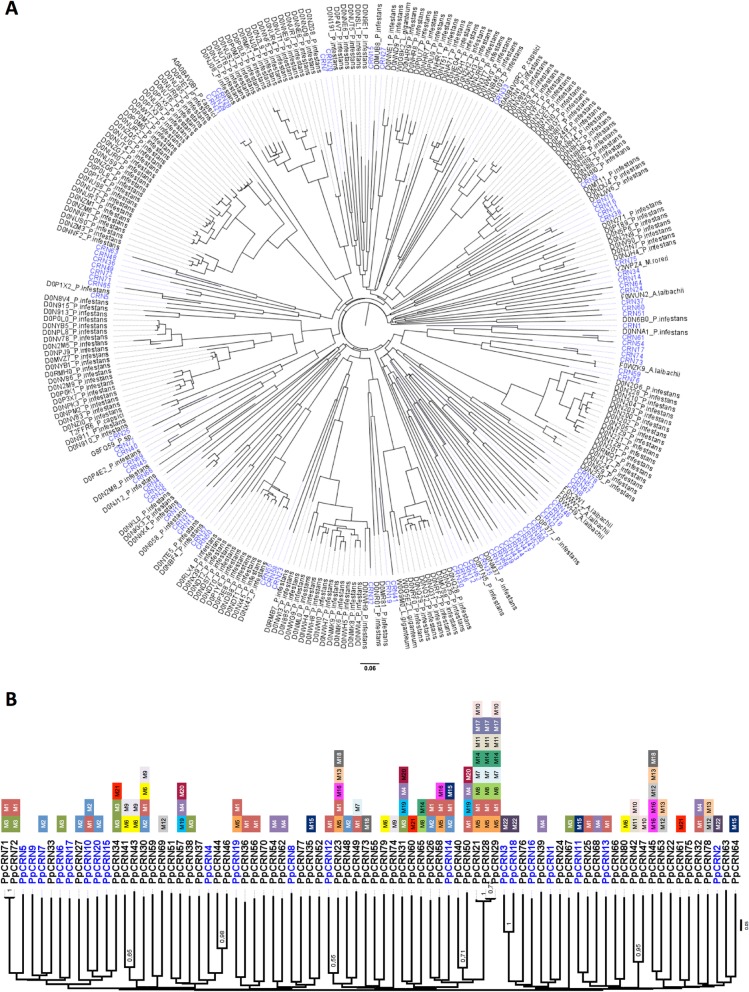
Phylogenetic analysis of predicted PpCRN from *P. parasitica* isolate ‘IAC_01/95.1’. **a** Sequences from *P. parasitica* isolate ‘IAC_01/95.1’ are shown in in blue, and sequences from other oomycetes identified in the Uniprot database are shown in black. Clusters were obtained according to the Neighbor-Joining method. Sequences obtained in the Uniprot are designated by their respective access number in the database and by species name. **b** Clustering based on the similarity of CRN motifs. In blue are shown the 20 PpCRNs selected for gene expression analysis. The variation of CRN motif are indicated by colored boxes named from M1 to M22

In order to verify the similarity between the identified putative PpCRN protein sequences and previously described CRNs, a Neighbour-Joining tree was predicted using all identified PpCRNs and CRN sequences from the Uniprot database (Fig. 3a). The wide distribution of PpCRN sequences over the tree shows a great sequence divergence between them. This distribution pattern was also followed by the *P. infestans* sequences used in the tree. In order to address this high divergence between PpCRN sequences, we searched for common motifs between them. Twenty-two motifs were predicted as present in at least three of the total sequences (Fig. 3b). The identification of distinct CRN motifs was named from M1 to M22 (Fig. 3b). Sequences and additional information of the 80 PpCRN candidates are shown in the Additional file 1.

**Fig. 4:**
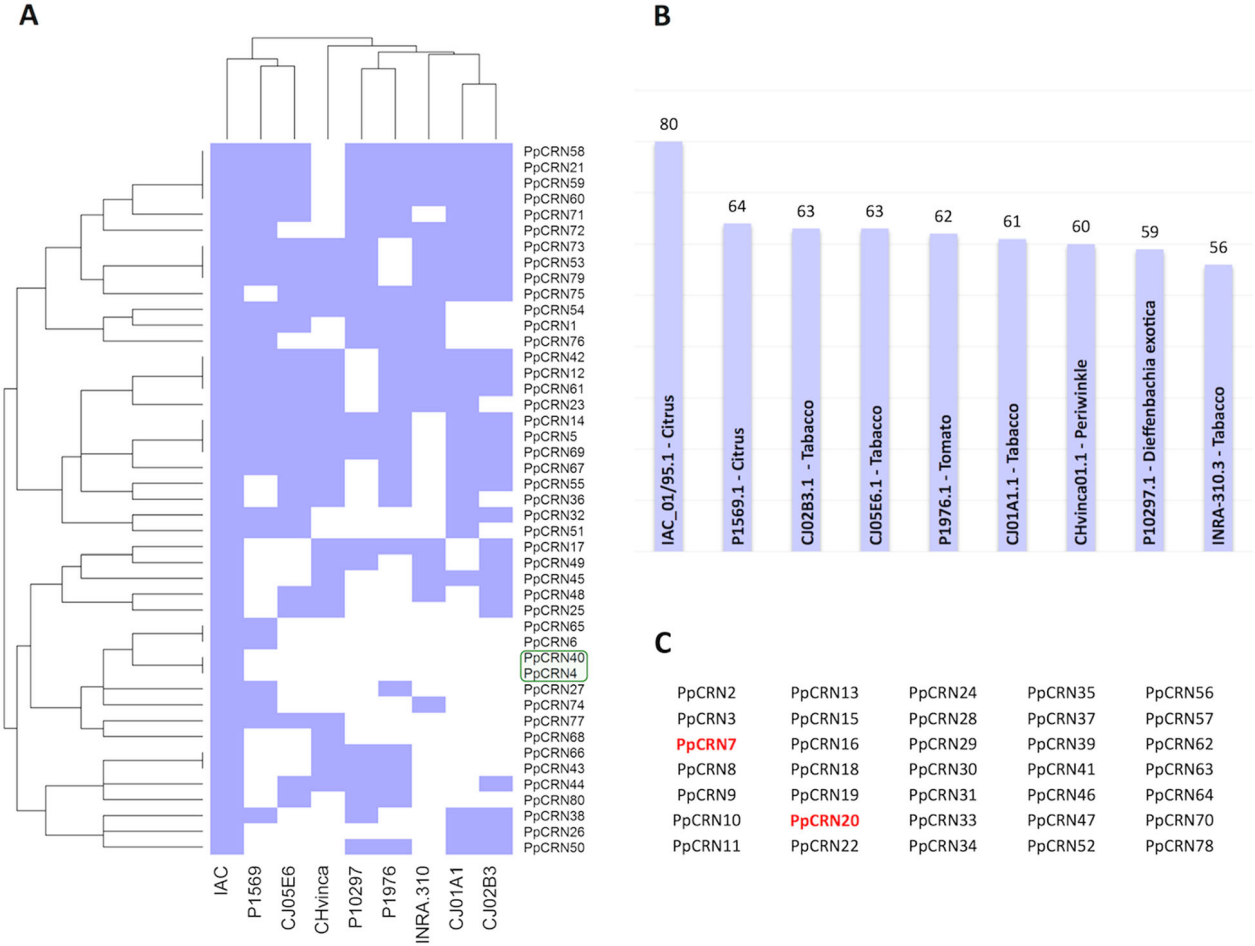
Genomic analysis of PpCRNs from diferent isolates of *P. parasitica*. **a** Dendogram of the distance between eight *P. parasitica* genomes, from isolates ‘P1569.1’, ‘P1976.1’, ‘INRA-310.3’, ‘P10297.1’, ‘CJ01A1.1’, ‘CJ05E6.1’, ‘CHvinca01.1’, ‘CJ02B3.1’, in relation to the ‘IAC_01/95.1’ genome. The green box marks PpCRN4 and PpCRN40 that are unique to the ‘IAC_01/95.1’ genome. **b** The 80 PpCRNs predicted in the isolate ‘IAC_01/95.1’ and the corresponding presence in the other eight genomes. **c** Identification of 35 predicted PpCRN that are found in all nine *P. parasitica* genomes. PpCRN7 and PpCRN20, which were further characterized, are highlighted in red

All 80 predicted *PpCRN*s from the isolate ‘IAC_01/95.1’ genome were used as query for searching homologous genes within other eight genomes from different isolates of *P. parasitica*, which are named ‘P1569.1’, ‘P1976.1’, ‘INRA-310.3’, ‘P10297.1’, ‘CJ01A1.1’, ‘CJ05E6.1’, ‘CHvinca01’,‘CJ02B3.1’ (Fig. 4). We have found similar PpCRNs in the genomes of other *P. parasitica* isolates by applying a Blastp search using the 80 PpCRN candidates protein sequences of the ‘IAC_01/95.1’ genome as query. This genomic approach revealed the distribution and proximity of genomes to the eighty PpCRNs of the ‘IAC_01/95.1’ isolate. Similar sequences to *PpCRN7* and *PpCRN20* were found in all nine *P. parasitica* genomes (Additional file 3: Fig. S1), whereas *PpCRN4* and *PpCRN40* are unique to the ‘IAC_01/95.1’ genome (Fig. 4a). Based on the 80 candidate *PpCRN*s, our analyses revealed that the closest genome to *P. parasitica* isolate ‘IAC_01/95.1’ is the isolates ‘P1569.1’ and ‘CJ05E6’ from citrus and tobacco (Fig. 4a). Most of the *PpCRN* candidates predicted in the isolate ‘IAC_01/95.1’ were also found in the other isolates, varying from 64 out of 80 candidates in the citrus isolate ‘P1569.1’ to 56 out of 80 candidates in the tobacco isolate ‘INRA-310.3’ (Fig. 4b). 35 predicted *PpCRNs* (43.75%) from isolate ‘IAC_01/95.1’ are also found in all other eight isolates, with at least one corresponding protein found in each *P. parasitica* isolate genome, presenting more than 95% identity and 50% coverage (Fig. 4c).

### Predicted *PpCRNs* are transcriptionally deregulated during *Citrus*-*P. parasitica* interaction

Twenty *PpCRN* candidates were selected for gene expression analysis during the plant-pathogen interaction between *P. parasitica* and two *Citrus* species (*C. sunki* and *P. trifoliata*). We chose these two citrus species because they have contrasting response to *Phytophthora parasitica* infection; *Citrus sunki* is susceptible and *Poncirus trifoliata* is resistant to this pathogen. These candidates, PpCRN1 to PpCRN20, were selected based on the presence of one or more of the following features: (i) presence of a secretion signal peptide; (ii) absence of transmembrane domains; (iii) differential gene expression in other plant-pathogen interaction studies; (iv) presence of conserved CRN domain; (v) nuclear or subcellular localization signals; (vi) sequence homology with effectors from other species; (vii) *PpCRN* gene located at the sparse regions of the genome.

**Fig. 5:**
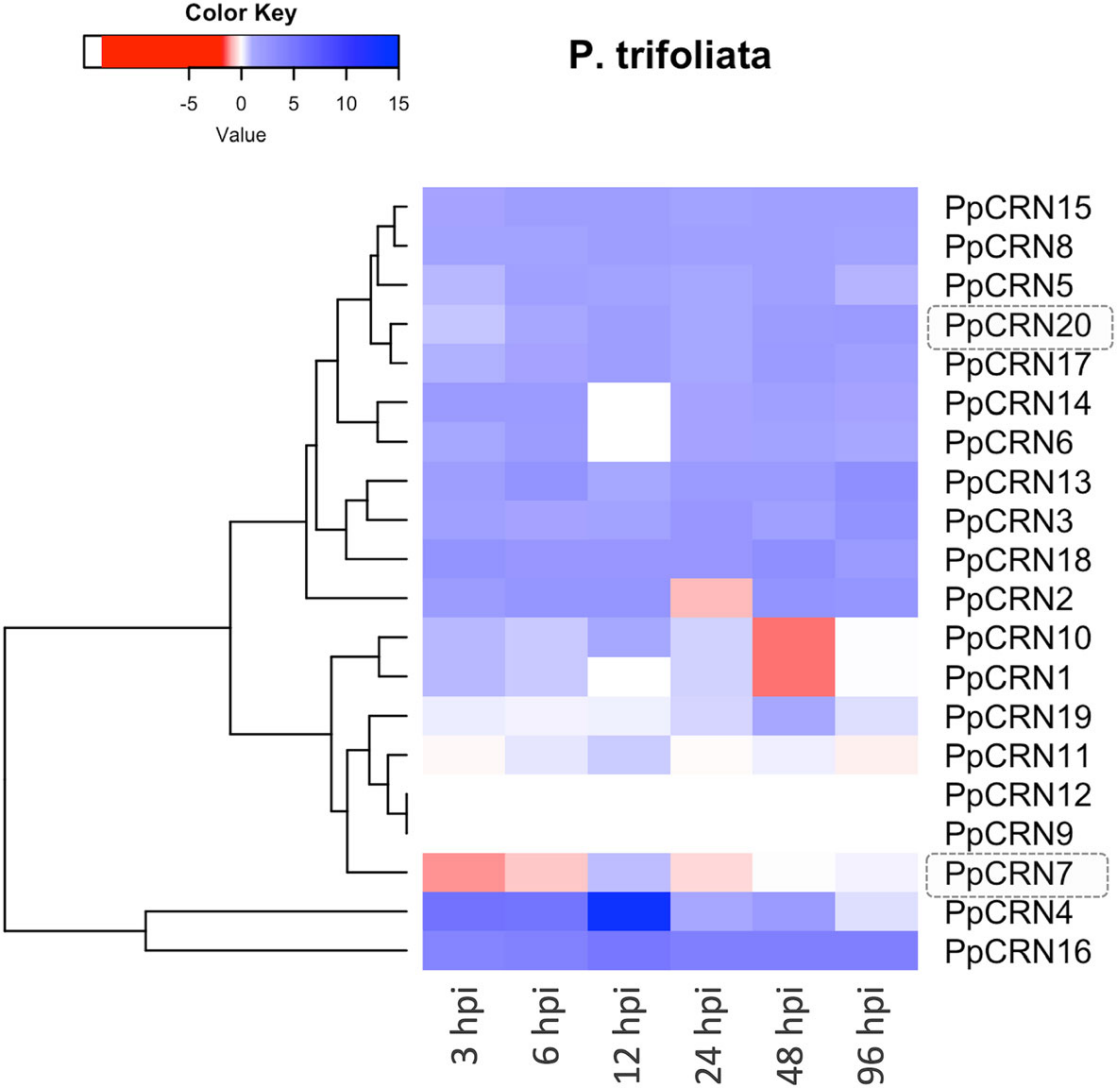
*PpCRN* gene expression analysis during *P. parasitica* interaction with *P. trifoliata*. Heat map shows gene expression levels of 20 predicted *PpCRN* effectors in *P. trifoliata* infected samples collected at 3, 6, 12, 24, 48, and 96 h post *P. parasitica* inoculation (hpi). The data are presented as expression relative to the reference genes S3A and UBC. The values ​​of gene expression are plotted in Log2. Up-regulating genes are indicated by the color red (> 1.0 - ≥ 15.0) and down-regulating genes by the red color (< 1.0 - ≤ − 15.0). PpCRN7 and PpCRN20, which were further characterized, are highlighted in grey

**Fig. 6:**
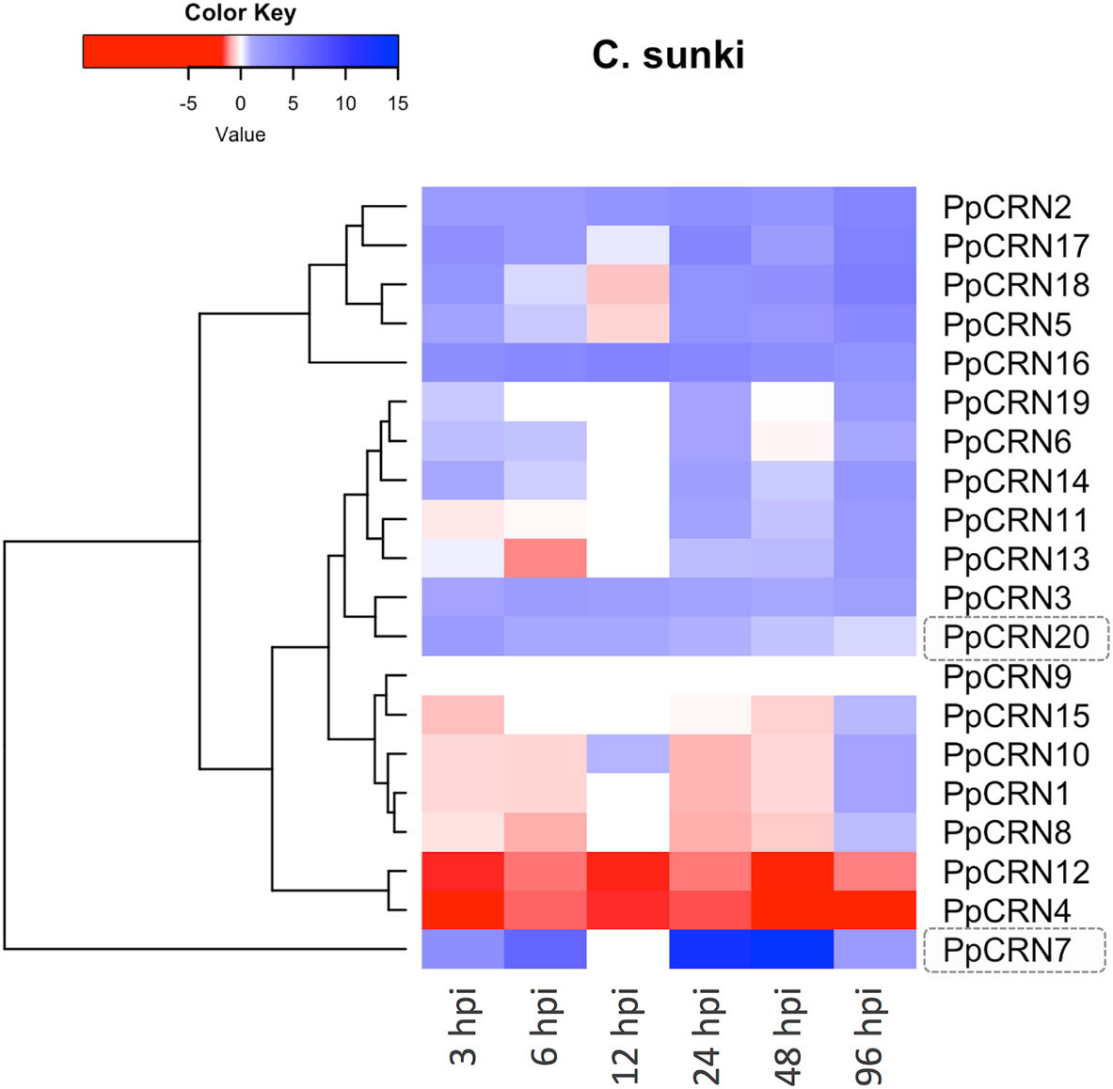
*PpCRN* gene expression analysis during *P. parasitica* interaction with *C. sunki*. Heat map shows gene expression levels of 20 predicted *PpCRN* effectors in *C. sunki* infected samples collected at 3, 6, 12, 24, 48, and 96 h post *P. parasitica* inoculation (hpi). The data are presented as expression relative to the reference genes S3A and UBC. The values ​​of gene expression are plotted in Log2. Up-regulating genes are indicated by the color red (> 1.0 - ≥ 15.0) and down-regulating genes by the red color (< 1.0 - ≤ − 15.0). PpCRN7 and PpCRN20, which were further characterized, are highlighted in grey

Gene expression analysis revealed an expressional dynamics of *PpCRN* effectors during the interaction of *P. parasitica* with the citrus plants. Our analysis showed that these *PpCRN* family members had their transcriptional levels altered, according to the citrus species and infective stage [11, 21] (Figs. 5, 6 and Additional file 4: Figure S2). Figure 5 shows that, in *P. trifoliata*, the vast majority of *PpCRNs* candidate genes were up-regulated along the time points, except for *PpCRN1*, *PpCRN7* and *PpCRN10* that were suppressed, at least in one time-point. *PpCRN4*, which is unique to the isolate ‘IAC 01/95.1’ genome, had the highest differential expression level detected among the *PpCRN* candidates, followed by *PpCRN16* and *PpCRN18*, both exhibiting high levels of transcripts. The candidates *PpCRN9*, *PpCRN11* and *PpCRN12*, showed constant expression levels throughout the time points analysed. In addition, *PpCRN7* expression were initially suppressed at 3 h post inoculation (hpi) and then returned steadily to basal levels at the 6 h time-point onwards, whereas *PpCRN20* were slightly induced 6 dpi onwards (Fig. 5).

In *C. sunki*, most of *PpCRN*s were transcriptionally induced (Fig. 6). However, *PpCRN4*, *PpCRN9* and *PpCRN12* transcripts were down-regulated at all time-points. *PpCRN8* and *PpCRN13* expression showed partial suppression in most of the time-points; at 96 hpi, an increase in their transcriptional levels were detected. Contrastingly, *PpCRN7* and *PpCRN20* expression were induced throughout the development of the disease, with *PpCRN7* showing the highest differential expressional level among all *PpCRN* candidates (Fig. 6). Recently our group published data showing that *P. parasitica* has the ability to recognize and regulate gene expression levels of effectors *CRN*, *RxLR*, *Elicitin*, *CBEL* and *NPP-1* over time and as a function of interaction with *C. sunki* and *P. trifoliata* [11].

### Functional characterization of the PpCRN7 and PpCRN20

Functional genomics were further taken to explore the potential role of two candidate PpCRN effectors, which are supposed to modulate cellular and molecular responses in host plants. Our in silico analyses identified that the candidate effectors *PpCRN7* and *PpCRN20* would be good candidates for characterization, as both showed: (i) secretory peptide signals; (ii) absence of transmembrane domains; (iii) genome location at the sparse regions; (iv) presence in all genomes of *P. parasitica* isolates herein analyzed, with a high degree of sequence identity; and (v) presence of known conserved *CRN* domains (Additional file 3: Figure S1 and Additional file 1).

Therefore, to reveal the functional role of *PpCRN7* and *PpCRN20*, a transient expression assay was carried out via agrotransformation in *N. benthamiana* leaves. The insertion of *PpCRN7* and *PpCRN20* transgenes in the plant-expressing vector pCambia1302 (Additional file 5: Figure S3A) was confirmed by gel eletroforesis after enzymatic digestion. The nucleotide fragments corresponds to the expected size of *PpCRN7* (430 pb) and *PpCRN20* (439 pb) (Additional file 5: Figure S3B). Furthermore, the expression of the proteins PpCRN7 and PpCRN20 *in plant* was confirmed by Western blotting (Additional file 5: Figure S3C). These constructs were then used to evaluate the effect of *PpCRN7* and *PpCRN20* to induce or supress HR in *N. benthamiana* leaves by co-expressing them along with the elicitin *INF-1* - a known cell death induction factor [22, 23]. The elicitin INF-1 is well known to induce HR in *Nicotianae* species. Therefore, it is commonly used in functional characterization studies of effectors [23].

### PpCRN7 enhances INF-1-induced HR response

**Fig. 7:**
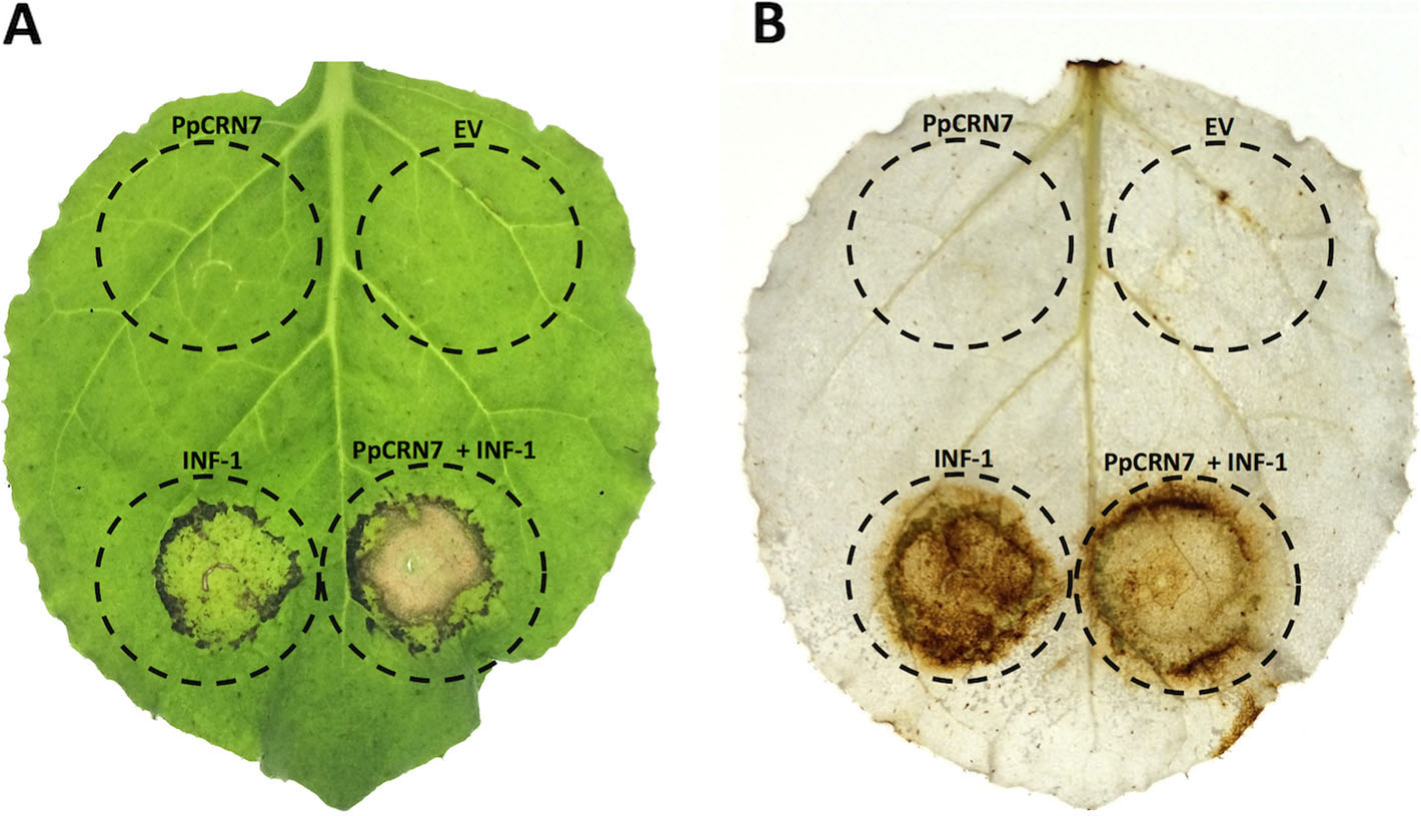
Transient expression of *PpCRN7* in *N. benthamiana* leaves. **a** *N. benthamiana* leaf agroinfiltrated with empty vector (EV), *PpCRN7*-containing vector, *INF-1*-containig vector, and *PpCRN7*- + *INF-1*-containing vectors. **b** DAB assay on *N. benthamiana*, as indicated in A, agroinfiltrated indicates H_2_O_2_ accumulation (brownish colour). Dots in circle represents the agroinfiltrated area

To test the effect of *PpCRN7* expression towards the HR mediated by the elicitin INF-1, we performed agrotransformation of plant expressing vectors containing (i) empty vector, (ii) *PpCRN7*, (iii) *INF-1*, and (iv) co-expression of *PpCRN7* + *INF-1* (Fig. 7a). No symptoms were observed in leaves infiltrated with the empty vector or *PpCRN7*-containing vector alone. However, *INF-1*-expressing leaves showed HR response, with evident tissue necrosis in the agroinfiltrated area, as also observed in leaves co-infiltrated with *PpCRN7* along with *INF-1* (Fig. 7a). Therefore, transient expression of *PpCRN7* + *INF-1* in *N. benthamiana* leaves revealed a synergistic activity of the CRN effector with the elicitin, as the HR response was intensified, leading to an anticipated and more prominent occurrence of PCD.

To verify if PpCRN7 effector also affects the release of reactive oxygen species (ROS) and oxidative burst, a biochemical and colorimetric assay was performed on *N. benthamiana* agroinfiltrated leaves (Fig. 7b). In this assay, the substrate DAB (3,3′-diaminobenzidine) is oxidized by hydrogen peroxide, to generate a dark brown precipitate, which allows the visual detection – the darker the tissue, the more ROS released. The *INF-1*-expressing area appears greatly dark, indicating that ROS were produced by still-living cells, whereas in the area co-infiltrated *INF-1* with *PpCRN7* showed mild dark staining, compared to the area expressing *INF-1* only (Fig. 7b). It suggests that cells in this area are already dead, due to the anticipation and amplification of ROS release and subsequent HR, driven by the synergistic activity of the effector PpCRN7 along with INF-1. Very likely, the biochemical target of the PpCRN7 effector is present down-stream the activation of ROS-release by INF-1, since, when alone, without INF-1, the PpCRN7 effector has no activity regarding release of ROS or induced PCD.

Additionally, we tested if the *A. tumefaciens* concentration, for the transient expression assay would be related to the observed HR amplification (Additional file 6: Figure S4). Agronfiltration solution were adjusted to an OD600 of 0.5 and 1.0 and used to co-infiltrate *PpCRN7* along with *INF-1*, and empty vector (EV) along with *INF-1*. The results were similar as *PpCRN7* enhanced *INF-1*-induced HR response, independent of *A. tumefaciens* concentration, confirming that PpCRN7 acts synergistically with INF-1 in the manipulation of plant defence mechanisms, which results in oxidative burst, programmed cell death and tissue necrosis (Additional file 6: Fig. 4).

### PpCRN20 suppresses INF-1-induced HR response

**Fig. 8:**
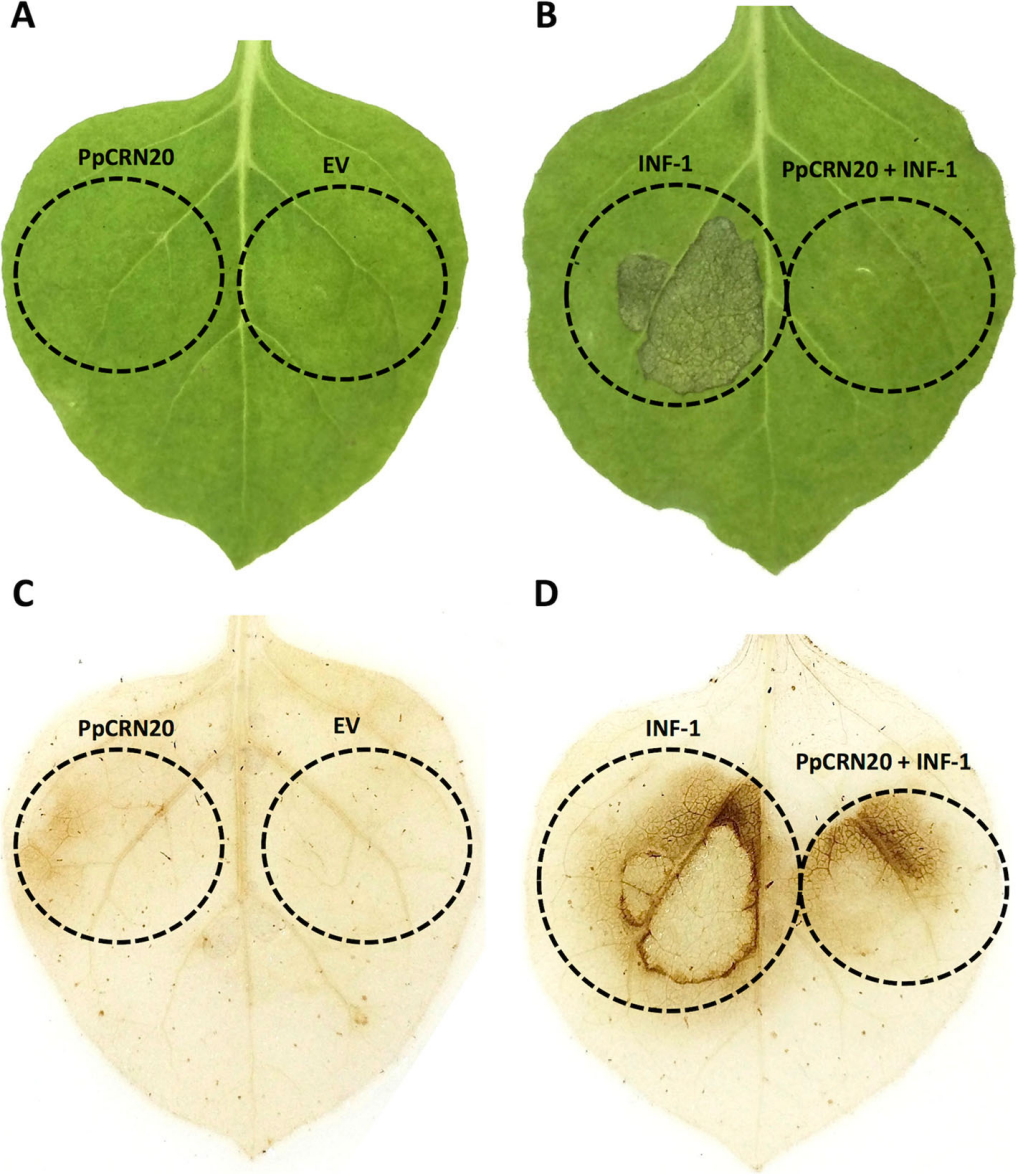
Transient expression of *PpCRN20* in *N. benthamiana* leaves. **a** *N. benthamiana* leaf agroinfiltrated with empty vector (EV) and *PpCRN20*-containing vector. **b** *N. benthamiana* leaf agroinfiltrated with *INF-1*-containig vector, and *PpCRN7*- + *INF-1*-containing vectors. **c** DAB assay on *N. benthamiana*, agroinfiltrated as indicated in A, indicates H_2_O_2_ accumulation (brownish colour). (**d**) DAB assay on *N. benthamiana*, agroinfiltrated as indicated in B, indicates H_2_O_2_ accumulation (brownish colour). Dots in circle represents the agroinfiltrated area

The same approach was carried out to test the effect of *PpCRN20* expression towards the HR mediated by INF-1. We performed agroitransformation of plant expressing vectors containing (i) empty vector, (ii) *PpCRN20*, (iii) *INF-1*, and (iv) *PpCRN20* + *INF-1*. No symptoms were observed in leaves infiltrated with the empty vector or *PpCRN20*-containing vector alone (Fig. 8a). However, as expected *INF-1*-expressing leaves showed HR response, with evident tissue necrosis in the area that was agroinfiltrated (Fig. 8b). The co-infiltration of *INF-1* along with *PpCRN20* presented a strong reduction on *INF-1*-induced symptoms. This result suggests that PpCRN20 acts as a suppressor of INF-1-induced HR response (Fig. 8b).

The DAB assay on *N. benthamiana* leaves showed no ROS production when expressing either an empty vector or *PpCRN20*-containing vector (Fig. 8c). However, leaves co-expressing *PpCRN20* along with *INF-1* showed a significant decrease in ROS production when compared to *INF1*-expressing site (Fig. 8d). The absence of tissue necrosis and decrease on ROS production indicates that PpCRN20 may act as HR suppressor.

### Transient expression of *PpCRN7* and *PpCRN20* increases *N. benthamiana* susceptibility

**Fig. 9:**
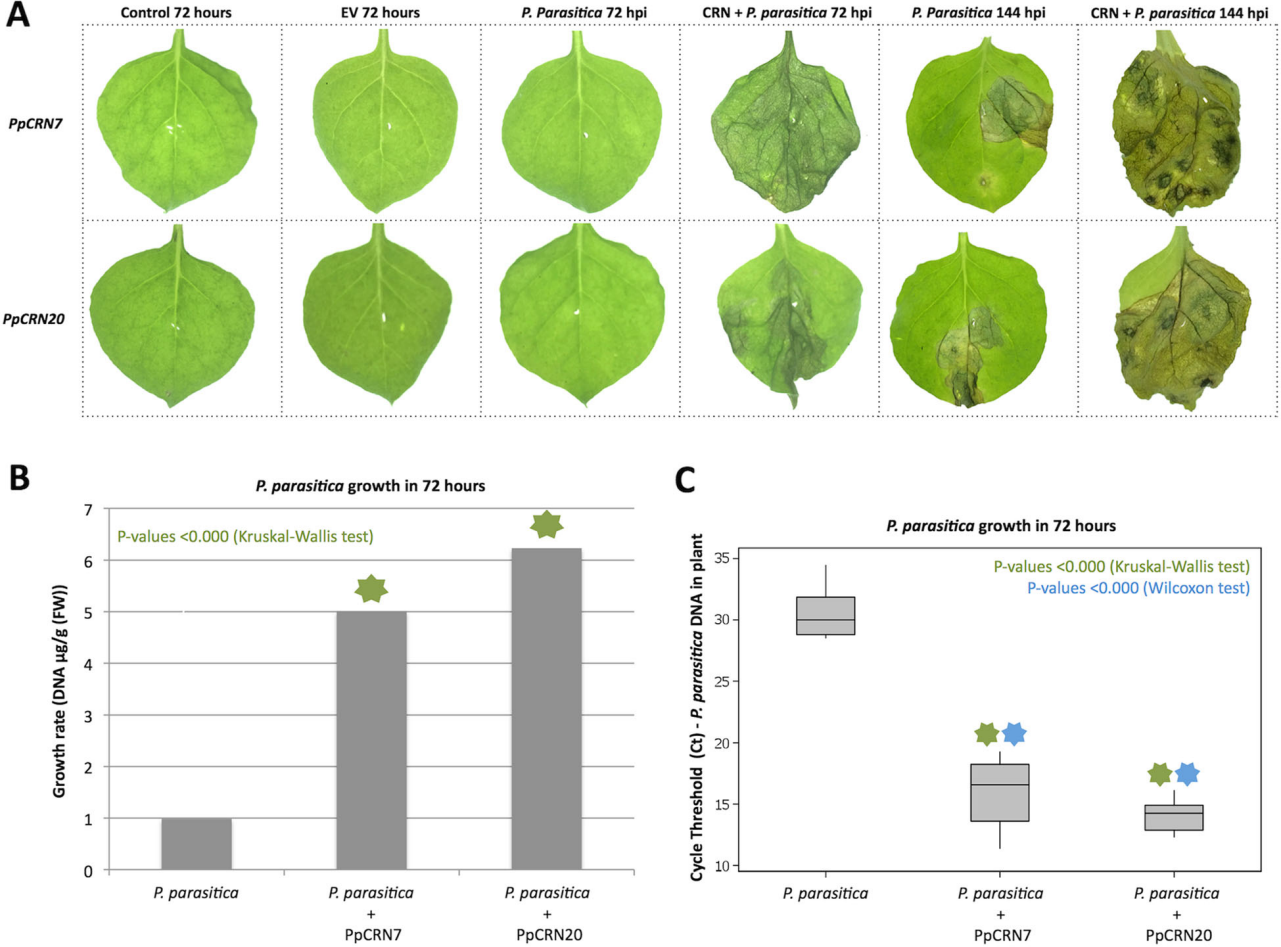
Transient expression of *PpCRN7* and *PpCRN20* on *N. benthamiana* leaves followed by *P. parasitica* infection. **a** *N. benthamiana* leaves transiently transformed with the effectors *PpCRN7* and *PpCRN20* and inoculated with 10^6^ zoospores of *P. parasitica*. Foliar tissues expressing *PpCRN7* and *PpCRN20* and inoculated with *P. parasitica* developed symptoms of severe wilting and necrosis at 72 and 144 h post inoculation (hpi). Controls and agroinfiltrated leaves with *PpCRN* genes without inoculation with *P. parasitica* zoospores, and leaves infected with *P. parasitica* zoospores did not develop visible symptoms. At 144 hpi all infected plants developed symptoms, however, these symptoms were stronger in plants that transiently expressed the *PpCRNs*. **b** Differences in DNA detection of leaves infected with *P. parasitica* (control) and *P. parasitica* + *PpCRN7*, *P. parasitica* + *PpCRN20* at 72 hpi are highly statistically significant. **c** The amount of genomic DNA of *P. parasitica* is statistically different for control plants, with values of: *p*-value < 0.000, indicated by the asterisk (Kruskal-Wallis test)

To understand the biological role mediated by PpCRN7 and PpCRN20 during the process of *P. parasitica* infection, *N. benthamiana* leaves were inoculated with zoospores of *P. parasitica*, 24 h after agrotransformation with *PpCRN7*- or *PpCRN20*-containing vectors. Leaves, transiently expressing *PpCRN7* and *PpCRN20*, inoculated with *P. parasitica* zoospores developed symptoms, measured at 72 and 144 h post inoculation (hpi), including severe wilt and tissue necrosis. Whereas *P. parasitica*-inoculated leaves, without *PpCRN* expression, showed symptoms only at 144 hpi (Fig. 9a). No symptoms were observed on leaves without *P. parasitica* inoculation expressing either an empty vector or any *PpCRN* (Fig. 9a).

*P. parasitica* genomic DNA from leaf tissues was quantified by RT-qPCR for samples collected at 72 hpi, to verify the growth rate and colonization of the oomycete, an indicative of *N. benthamiana* susceptibility (Fig. 9b). Significant differences were detected in *P. parasitica*-inoculated plants previously infiltrated with any *PpCRN*-expressing vectors, and only inoculated plants (Fig. 9c). Higher amount of *P. parasitica* genomic DNA was found in leaves expressing *PpCRN7* and *PpCRN20* compared to leaves without *PpCRN* expression (Fig. 9b,c). Significant differences were detected in transformed and subsequently inoculated samples, when compared to only inoculated plants at 72 hpi. Leaves expressing PpCRN20 presented the highest significant amount of *P. parasitica* genomic DNA, followed by leaves expressing PpCRN7.

## Discussion

Plant-pathogen interaction involves a complex and intricate network of attack versus defense strategies. The pathogen aims to invade the host cells to obtain nutrients and complete its life cycle. On the other hand, the plant uses defense systems to contain the infection and ensure its own survival. The attack strategy of *Phytophthora* species relies on the secretion of effectors, in particular CRN effectors that modulate the interaction with the host plant defences and enable the infection and colonization processes [18, 19]. Although CRN effectors are predominantly associated with the appearance of necrosis, there are a number of CRN effectors that can inhibit cell death, triggered by PAMPs [18].

In natural populations, changes in the frequency of effectors, driven by the resistance structure of the host population, are complemented by genetic drift, which may generate marked differences as a consequence of the survival or extinction of individual pathogen strains in a given geographical condition or space [24, 25]. On the other hand, gene flow or migration of pathogen populations may lead to the establishment of new populations or the introduction of new virulence combinations in existing populations [26]. The number of candidate *PpCRN* effector genes predicted was different in each genome of *P. parasitica* isolates, as well as the LxLFLAK-like motifs varied among the isolates [15, 18]. However, 35 predicted *PpCRN*-encoding genes were found conserved among all nine isolates. We believe that the PpCRN effectors might be important for *P. parasitica* pathogenicity during plant-pathogen interaction considering the complexity and diversity of these effectors in *P. parasitica* isolates and their corresponding ecosystems. On the other hand, the predicted *PpCRN4* and *PpCRN40* genes that were exclusively found in the genome of the isolate ‘IAC01/95.1’ might potentially represent an adaptation to the ecosystem that it was originated.

Based on the 80 predicted *PpCRN*s, the isolates ‘IAC_01/95.1’ and ‘P1569’ were the closest related *P. parasitica* isolates. Both of them were isolated from citrus plants. The lower genetic variability of these two isolates may be related to an adaptation to infect the same host. Additionally, this genome proximity could be also related to geographical factors, considering that the three closest related isolates (‘IAC_01/95.1’, ‘P1569’ and ‘CJ05E6’) belong to the same American continent and probably evolved from the same ancestor.

The protein primary structure of 80 CRN effector candidates from *P. parasitica* was identified in silico and presented distinct motifs of LxLFLAK. This complexity and diversity can be attributed to the hemibiotrophic lifestyle of *P. parasitica*, as well as the adaptive and co-evolutionary forces that emerge from the great variety of host plants, which represents more than 250 genera [27]. This diversity found in PpCRNs primary structure can be, therefore, explained by the fact that effector molecules are modular, and likely to undergo changes in their sequences under selective pressure. These changes in gene sequence may inactivate the effector protein or rather provide a new function, increasing the pathogen adaptation to environmental challenges (Haas et al., 2009). The diversity of N-terminal and C-terminal portions of PpCRN sequences is consistent with results reported for a range of microorganisms, including *Rhizophagus irregularis, P. capsici, Batrachochytrium dendrobatidis, Albugo laibachi, A. cândida, Pythium ultimum, Hyaloperonospora arabidopsidis, P. infestans, Aphanomyces euteiches, P. sojae, P. ramorum* [15, 16, 28–35].

*PpCRN7* and *PpCRN20* are found in all *P. parasitica* isolates herein investigated, with a high degree of protein sequence conservation and located at the sparse regions, suggesting that both effectors may play a crucial role in the interaction of *P. parasitica* and host plants, likely modulating plant defence mechanisms. These two effector proteins have typical CRN motifs, the LxLFLAK-like motif, “LYLATK” in PpCRN7 and the “LFLAK” in PpCRN20, as observed in different species of *Phytophthora spp*. [15, 29], followed by “DI” and “DWL” motifs at the N-terminal region.

In silico analysis and further functional characterization assays unveil the biological role of the two CRN effectors from *P. parasitica*. To investigate the role of these two PpCRNs effectors, a functional genomic and proteomic study was performed to explore the information obtained from *P. parasitica* genomes, and to evaluate the modulation of *N. benthamiana* defence responses, based on activation or suppression of HR response. PpCRN7 effector shows synergy with *P. infestans* elicitin INF-1 by anticipating and amplifying HR and PCD in *N. benthamiana* agroinfiltrated leaves. This effector-induced cell death promoted susceptibility, and similar results have already been reported for PiCRN8 and PsCRN63, effector proteins from *P. infestans* and *P. sojae*, respectively [36, 37]. The activity of the effector PsCRN63 is linked to disruption of the plant ROS homeostasis, by directly interacting with host catalases [38]. Our results suggest that the PpCRN7 effector is targeting/regulating a compound that is present down-stream the activation of ROS-release, however the exact nature of this target still remains to be elucidated. In a trophic point of view, it makes sense for *P. parasitica* to have/deploy an effector that can intensify the HR responses only after there was some PAMP or effector recognition, followed by defense activation by the host. That will result in a greater necrotic area that will benefit the necrotrophic stage of the pathogen. A strong HR response may fail to kill hemibiotrophic pathogens such as *P. parasitica*, as these can feed on dead tissue. It has been reported [11] that a strong defense response of *Citrus sunki* infected with *P. parasitica* led to the activation of a vigorous HR that was not sufficient to kill the pathogen, but rather increased its colonization [11].

Multiple effectors might act in the same pathways and only the most efficient effector might prevail and manifest the disease symptoms. PpCRN7 has an additive effect on INF-1 function, enhancing HR.

Conversely, PpCRN20 effector suppresses HR response, which resulted in a decrease of ROS accumulation in agroinfiltrated *N. benthamiana* leaves. Hence, it suggests that PpCRN20 might be an important effector used by *P. parasitica* to combat the plant defences. Several CRN effectors have been shown to suppress elicitor-triggered plant cell death [39, 40]. For instance, *P. sojae* PsCRN70 and PsCRN115 suppress PCD by, decreasing H_2_O_2_ accumulation and down-regulating defense-associated genes, including *PR1b*, *PR2b*, *ERF1* and *LOX* genes [19] and interacting with plant catalases to inhibit PCD via ROS accumulation [20]. Similarly, the *P. parasitica* RxLR effector, PpRxLR2, was able to completely inhibit the INF-1 induced cell death in *N. benthamiana* leaves [22].

*P. parasitica* is a hemibiotrophic pathogen and as such has the ability to feed on living or dead tissues. Several authors pointed that the hemibiotrophic lifestyle of *P. parasitica* can be obeserved in two temporal phases, an initial biotrophic one and later a necrotrophic phase [41–43]. Based on that, we propose the biological model of the interaction between *P. parasitica* and plant hosts, which includes the temporal and functional activity of PpCRN7 and PpCRN20 effectors.

**Fig. 10:**
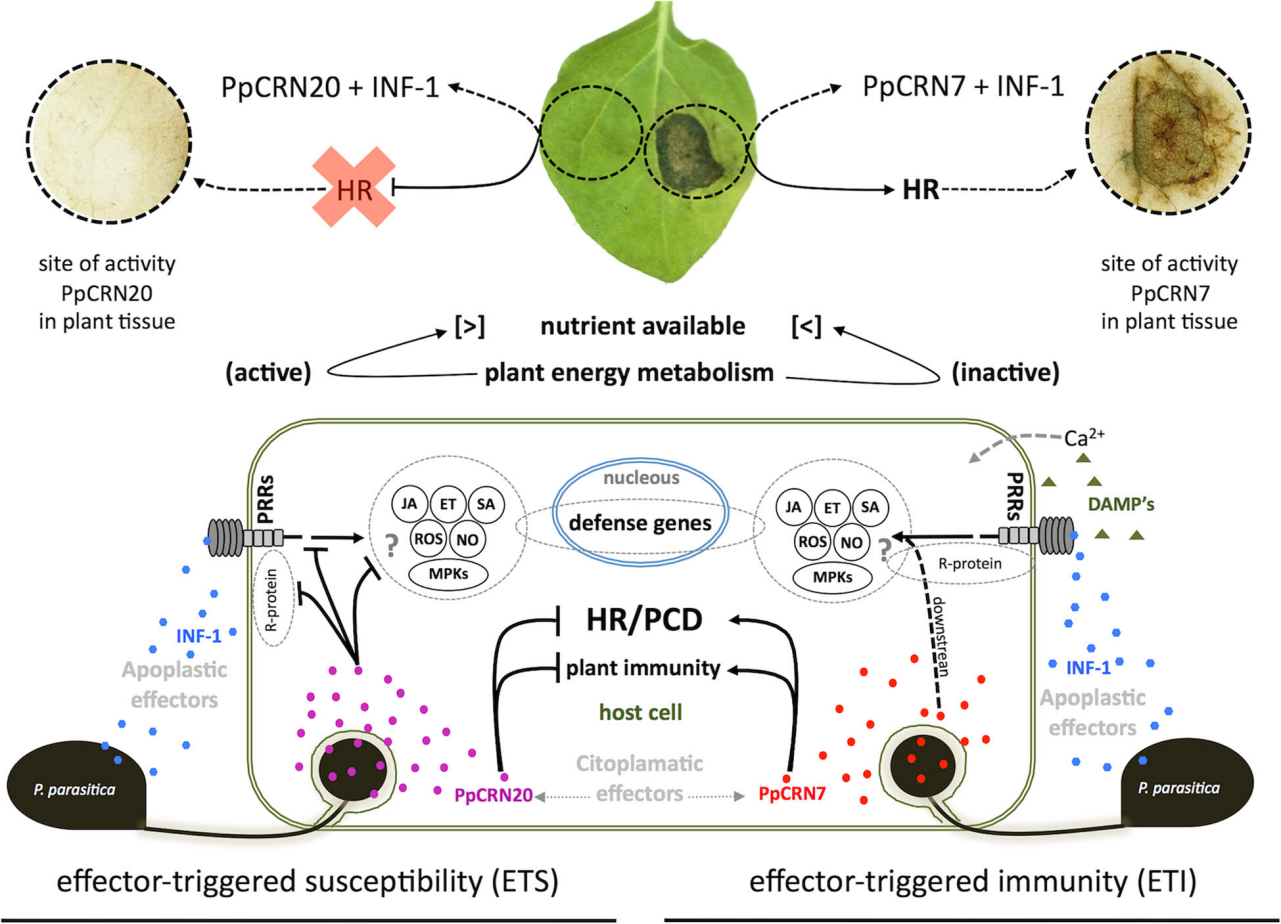
Schematic model of the interaction between *P. parasitica* and host plants, including the biological role of PpCRN7 and PpCRN20 effectors. On left, figure represents the PpCRN20 acting on hypersensitive response (HR) and cell-death suppression. On right, figure represents the PpCRN7 acting synergistically with the elicitin INF-1 to promote and potentiate HR and subsequent PCD. The sites of activity of the effectors PpCRN20 and PpCRN7 are highlighted by DAB assay

Our hypothesis is that PpCRN20 acts in initial stages of infection, playing an important role by suppressing HR and PCD in plants, thus favoring the infection and colonization of plant living tissues. In this biotrophic phase, plant cells provide durable and renewable nutrients, the integrity of photosynthetic and metabolically active plant tissues would favor *P. parasitica* energetic fitness and cell cycle and will contribute to the colonization establishment. Later on infection, the plant physiological homeostasis might be already compromised, some individual cells or tissues may be damaged, leading to host recognition of damage-associated molecular patterns (DAMPs) and activation of defense responses, including HR. The pathogen colonization process is completed with the activity of PpCRN7, that potentiate HR, and subsequently, PCD in plant tissues. This significant increase of dead plant tissues provides a final dose of energetic molecules that can be used by the pathogen to complete its life cycle. Our hypothesis is summarized in the Fig. 10.

## Conclusions

PpCRN7 and PpCRN20 are associated with the aggressiveness of *P. parasitica* as well as enhancing susceptibility of plants, therefore their mechanisms are now targets of biotechnological studies aiming to disrupt the activity of these effectors breaking pathogen’s aggressiveness and to promote plant resistance. Another approach will be to identify and edit the plant molecular targets or susceptibility genes that these effectors act upon, also resulting in resistance.

## Methods

### Oomycete growth conditions

*Phytophthora parasitica* isolate ‘IAC_01/95.1’ (stored in the microorganisms collection of the IAC-Cordeiropolis-SP Brazil) was grown in a carrot-agar medium, at 25 °C in the dark. Sporangia development was performed according to Máximo et al. (2017) [11, 21, 22]. Briefly, after colony reaching up to 80% of the plate capacity, sporangia development were induced by pouring sterile water on plate, replacing water daily for one week. To induce zoospores release, plates were incubated at 4 °C, in the dark, during one hour. Zoospore suspension was collected and adjusted to a concentration of 10^5^ zoospores/mL. The amount of zoospores in suspension was counted in a Neubauer chamber.

### Plant infection and agroinfiltration assay

Seeds of “Sunki” mandarin (*Citrus sunki* Hort. Ex Tan.) and trifoliate orange (*Poncirus trifolita* (L.) Raf. cv, Rubidoux) were obtained from the collection of the active citrus germplasm bank of Centro de Citricultura Sylvio Moreira/Instituto Agronômico de Campinas (CCSM/IAC), Cordeirópolis, São Paulo, Brazil. Seeds of *Citrus sunki* and *Poncirus trifoliata* were germinated in sterile soil. Three months after germination, the substrate was carefully removed and the seedlings transferred to Falcon tubes containing 50 mL of distilled water. A suspension of 10^5^ zoospore/mL was added to the recipient and sealed with Parafilm to reduce losses through evaporation. Plants were incubated in growth chamber at 20 °C under photoperiod of 12 h (250 μmol m-2 s-1). Six biological replicates for each treatment were used and the assay repeated two times. Plant roots samples were harvested 0, 3, 6, 394 12, 24, 48 and 96 h post inoculation (hpi) to analyse the expression of putative *PpCRN* genes from *P. parasitica*. These time points were selected in accordance to the *P. parasitica* hemibiotrophic life style, described previously [11, 21, 22]. *N. benthamiana* seeds were obtained from the seed bank collection of Centro de Citricultura Sylvio Moreira/Instituto Agronômico de Campinas (CCSM/IAC), Cordeirópolis, São Paulo, Brazil. The agroinfiltration assays were carried out with plants of 4–6 weeks old of *N. benthamiana*. The plants were grown under 16/8 h (light/dark) photoperiod at 22–25 °C, 60% humidity with 200 μmol m − 2 s − 1 illumination during the day period. Young and fully expanded leaves were used.

### Identification and annotation of *P. parasitica* candidate *CRN* effector genes

Candidate CRN effector sequences from *P. parasitica* isolate ‘IAC_01/95.1’ (PpCRN) were identified as following: (i) identification of protein sequences that belongs to CRN family deposited in the Uniprot database; (ii) search for homologous sequences in the genome of isolate ‘IAC_01/95.1’, using sequences identified in the Uniprot database as query for BLAST analysis (BlastP e-value>1e-05); (iii) PpCRN protein sequences were analyzed for the identification of conserved domains, and for the presence/absence of transmembrane domains using the TMHMM 2.0 software (http: //www.cbs.dtu.dk / services / TMHMM /); (iv) identification of peptide signals by SignalP 3.0 software (http://www.cbs.dtu.dk/services/SignalP-3.0/), and for prediction of the subcellular localization, using the WoLF PSORT software (http://wolfpsort.org/); and (v) PpCRN protein sequences were aligned and grouped by similarity using the cluster analysis and the neighbor-joining method. These analyses were also performed using public available sequences under the “*Phytophthora parasitica genome initiative*”, corresponding to the genomes: ‘P1569.1’, ‘P1976.1’, ‘INRA-310.3’, ‘P10297.1’, ‘CJ01A1.1’, ‘CJ05E6.1’, ‘CHvinca01.1’, ‘CJ02B3.1’ in order to identify the number of putative CRNs candidates in these isolates. The description of all *P. parasitica* putative CRN protein sequences is present in the Additional file 1. The CRN candidate sequences, namely PpCRN (*Phytophthora parasitica* Crinkler) were followed by the numerical order of appearance in our in silico analysis.

The presence of similar CRN effectors in the genomes of other *P. parasitica* isolates was verified applying a Blastp search using the protein sequences of the 80 PpCRN candidates from ‘IAC_01/95.1’ genome as query. For this analysis, a minimum protein sequence identity and coverage values ​​of 95 and 50%, respectively, were set. The number of genes, frequency, geographical origin of the *P. parasitica* isolates and hosts are shown in Table 1.

**Table 1:** Genomes publicly available of *Phythophthora parasitica*. Different isolates of *P. parasitica*, whose genomes are sequenced and publicly available, indicating their number of genes, geographic origin, and their respective host plants

Isolate/Genome*	Number of genes	%	Origin	Host
P1569.1	23.159	100	California	Citrus
P1976.1	23.127	99.8	California	Tomato
INRA-310.3	23,121	99.8	Australia	Tobacco
P10297.1	23.114	99.8	Florida	*Dieffenbachia exotica*
CJ01A1.1	23.061	99.5	Virginia	Tobacco
CJ05E6.1	21.812	94.1	Virginia	Tobacco
IAC_01/95.1	21.789	94.0	São Paulo	Citrus
CHvinca01.1	21.679	93.6	Virginia	Periwinkle
CJ02B3.1	21.085	91.0	Virginia	Tobacco

*Phytophthora parasitica Assembly Dev initiative, Broad Institute (broadinstitute.org)

### Genome architecture of *P. parasitica*

Distribution of 80 predicted *PpCRN* effectors in the ‘IAC 01_95’ genome was carried out by genome architecture analysis, based on two dimensional method of binary data using R software. This method is flexible a combines genome architecture heatmaps with scatter plots of the genomic environment and the pool of selected genes [44].

### Plasmid design and agro-transformation

Gene sequences of *PpCRN7* and *PpCRN20* fused to a 3xHA tag were obtained by gene synthesis and cloned in the vector pCambia 1302 (Additional File 2 and Additional file 5: Figure S3). The recombinant plasmids were sequenced and used for transformation of *Agrobacterium tumefaciens* GV3101. Transformed agrobacterium were cultivated in LB agar plates supplemented with kanamycin 50 μg/mL and rifampicin 50 μg/mL for 2–3 days at 28 °C. From a single colony on LB plate, we prepared a pre-inoculum of agrobacterium in 3 mL liquid LB medium with antibiotics for 24 h at 28 °C and 200 RPM. 40 μL of the pre-inoculum was taken to get an inoculum of 10–15 mL YEB (Agrobacterium growth medium) with same antibiotics, 2 μM acetosyringone and 10 mM MES and growth overnight till it reached the final OD of approximately 1. The bacteria in the medium were precipitated (4000×g, 10 min), and the pellet resuspended in MMA medium 20 g sucrose, 5 g MS salts, 1.95 g MES, pH adjusted to 5.6 with NaOH, and 1 ml acetosyringone/L). Final OD_600nm_ was adjusted to 0.5–1.0. The cells were incubated at room temperature for 3 h. Infiltrations were performed with 1 mL syringe by pressing the needleless syringe on the underside of the leaf. Plants were incubated at 25 °C in an 12 h photoperiod. Symptoms were observed 2–7 days after infiltration. *PpCRN7*- and *PpCRN20*-containing vectors were co-infiltrated with *INF-1*-containg vector. These assays were repeated three times.

### Detection of reactive oxygen species (ROS) in plant tissue to evaluate HR

For detecting ROS, specifically H_2_O_2_ in leaves of *N. benthamiana*, it was carried out the DAB assay at 5 days post infiltration according to Thordal-Christensen et al. (1997) [45] and Salzer et al. (1999) [46]. The stained leaves were analyzed by light-microscopy.

### *P. parasitica* inoculation and quantification of their DNA in plant to asses disease development

To evaluate the activity of the *PpCRN7* and *PpCRN20* during the colonization and development of *P. parasitica* in *N. benthamiana* leaves, we performed agroinfiltration of candidate effectors followed by zoospores inoculation (24 h after agroinfiltration). 1 × 10^6^ *P. parasitica* zoospores were inoculated in agroinfiltrated spots. Leaves agroinfiltrated with empty vector used as controls. Three biological replicates were used for each treatment. The leaves were harvested 72 hpi, after emergence of symptoms to evaluate the amount of genomic DNA of *P. parasitica*. Fresh mycelia from carrot solid medium (100 mg) was grounded in liquid nitrogen and preceded to DNA isolation and purification using DNeasy plant mini kit (Qiagen). DNA was further purified with Wizard® Kit (Promega) according to the manufacturer’s recommendation. DNA samples were evaluated for purity and concentration by ultraviolet spectroscopy (NanoDrop 8000, Thermo Scientific). RT-qPCR was performed with a mixture of diluted DNA (1:20 H_2_O), GoTaq® Real-Time qPCR (Promega) and 10 pmol of each primer PN5b (5’GAACAATGCAACTTATTGGACGTT3’) and PN6 (5’ AACCGAAGCTGCCACCCTAC3’) (ITS regions) in a final volume of 20 μL [47]. Reactions were carried out with the following thermal cycler program, an initial denaturation at 95 °C for 10 min and 40 cycles: 95 °C 15 s, 62 °C 60s. The Ct values were plotted in a standard curve generated from a sample with known DNA concentration, to determine the concentration of DNA in the evaluated sample. The concentration of DNA in the standard curve ranged from 1 pg to 10 ng DNA mL − 1. The results were analysed with Kruskal-Wallis [48] and Wilcoxon test [49].

### Protein extraction and western blot analysis

Leaf tissues of *N. benthamiana* were grounded to a fine powder under liquid nitrogen using a sterile mortar and pestle and rinsed with extraction buffer (Hepes 50 mM, KCL 150 mM, EDTA 1 mM, Triton X-100 0.1%, with pH adjusted to 7.5 with KOH) supplemented with 1 mM DTT and protease inhibitor. Total protein extracts were transferred to 15% SDS-polyacrylamid gels, and the pattern of bands further analyzed. Proteins were transferred to a nitrocellulose membrane, and membranes were washed in PBST (PBS with 0.1% Tween 20) during 2 min and blocked during 30 min in PBST-BSA (PBS with 0.1% Tween 20 and 3% bovine serum albumin (BSA)). Rabbit anti-HA monoclonal antibody was added to the PBST-BSA buffer and incubated for 20 min using the method SNAP i.d. 2.0 Protein Detection System, followed by washing steps with PBST (three times). Membranes were then incubated in PBST-BSA in addition to the chemiluminescent secondary antibody reactive to luminol. The immunoreactive bands were detected on x-ray film by enhanced chemiluminescence with luminol substrate and subsequently photoregistrated.

## Supplementary information

**Additional file 1.** Sequences and additional information of the Crinkler candidates .xls (1,8 MB).

**Additional file 2.** Nucleotide sequences of the CRN’s effectors PpCRN7 and PpCRN20 used for transformation .pdf (67 KB).

**Additional file 3. **Fig. 1**:** Sequence alignment of amino acid sequences of PpCRN7 and ppCRN20 from *P. parasitica* isolates .TIFF (18,7 MB).

**Additional file 4. **Fig. 2**:** *PpCRN* gene expression analysis during *P. parasitica* interaction with *C. sunki* and *P. trifoliata* .TIFF (20,4 MB).

**Additional file 5. **Fig. 3**:** Plant-expressing vector used for the functional characterization of PpCRN genes and validation of protein expression .TIFF (12,7 MB).

**Additional file 6. **Fig. 4**:** Transient expression of *PpCRN7* in *N. benthamiana* leaves .TIFF (11,4 MB).

## Data Availability

All data generated or analysed during this study are included in this published article and its supplementary information files. The analysis were performed using public sequences available under the [*Phytophthora parasitica* genome initiative], [https://www.ncbi.nlm.nih.gov/assembly/?term=phytophthora%20parasitica] and [UniProt] [https://www.uniprot.org/].

## Abbreviations

°C: degrees Celsius
Anti-HA: anti-Hemagglutinin (antibody)
μg: microgram
μL: Microliter
μM: micromolar
Ca2 +: Calcium ion
CBEL: Cellulose-Binding, Elicitor and Lectin activity
CHvinca01.1: *Phytophthora parasitica* isolate/genome
CJ01A1.1: *Phytophthora parasitica* isolate/genome
CJ02B3.1: *Phytophthora parasitica* isolate/genome
CJ05E6.1: *Phytophthora parasitica* isolate/genome
CRN: effector of the Crinkler family
DAB: 3,3′-diaminobenzidine
DAMPs: Damage-Associated Molecular Patterns
DI: motif of protein Crinkler
DTT: DL-Dithiothreitol
DWL: motif of protein Crinkler
EDTA: Ethylenediaminetetraacetic acid
*ERF1*: ethylene response factor 1
ET: Ethylene
ETS: effector-triggered susceptibility
EV: Empty Vector
FW: fresh weight
g: gram
GV3101: *Agrobacterium tumefaciens* strain
h: hours
H_2_O_2_: Hydrogen peroxide
hpi: hours post inoculation
HR: Hypersensitivity Response
HVLVXXP: motif of protein Crinkler
IAC_01/95.1: *Phytophthora parasitica* isolate/genome
INF-1: elicitin 1 of *Phytophtora infestans*
INRA-310.3: *Phytophthora parasitica* isolate/genome
JA: Jasmonate
KCL: Potassium chloride
KOH: Potassium hydroxide
L: Litre
LB: Luria-Bertani medium Leucine–Alanine–Lysine
LFLAK: LxLFLAK-like motif
*LOX*: (Protein Coding), Lysyl Oxidase
LxLFLAK: motif of protein Crinkler Leucine–any amino acid–Phenylalanine
LYLATK: LxLFLAK-like motif
MES: MES Medium
mg: Milligram
mL: millilitre
mM: millimolar
MMA: MMA Medium
MPKs: Mitogen-Activated Protein Kinases
NaOH: Sodium hydroxide
ng: nanogram
NO: Nitric oxide
NPP-1: Necrosis-inducing *Phytophthora* Protein 1
OD: Optical Density
P10297.1: *Phytophthora parasitica* isolate/genome
P1569.1: *Phytophthora parasitica* isolate/genome
P1976.1: *Phytophthora parasitica* isolate/genome
PAMPs: Pathogen-Associated Molecular Patterns
pb: base pair
PBS: Phosphate-buffered saline
PCD: Programmed Cell Death
pg: picogram
pH: potential of hydrogen
PiCRN8: *Phytophthora infestans* Crinkler number 8
PN5b: pimer pair name (*Phytophthora nicotianae* 5b)
PN6: pimer pair name (*Phytophthora nicotianae* 6)
PpCRN: candidate Crinkler effectors in *Phytophthora parasitica* genomes
*PpCRN*: genes encoding Crinkler effector proteins in the genome of *P. parasitica*
PpCRN20: *Phytophthora parasitica* Crinkler effector proteins number 8
PpCRN7: *Phytophthora parasitica* Crinkler effector proteins number 7
*PR1b*: Pathogenesis-related protein 1b
*PR2b*: Pathogenesis-related protein 2b
PRRs: Pattern Recognition Receptors
PsCRN115: *Phytophthora sojae* Crinkler number 115
PsCRN63: *Phytophthora sojae* Crinkler number 63
PTI: Pattern-Triggered Immunity
ROS: reactive oxygen species
RPM: rotations per minute
RT-qPCR: Quantitative reverse transcription Polymerase Chain Reaction
RxLR: effector of the RxLR family
S3A: 40S ribosomal protein S3a
SA: Salicylic acid
SDS: sodium dodecyl sulfate
UBC: Ubiquitin-conjugating enzyme
YEB: Yeast Extract Beef medium
μmol m − 2 s − 1: Micromole per second and square meter
