# Supplementary figures and images for: PpCRN7 and PpCRN20 of Phythophthora parasitica regulate plant cell death leading to enhancement of host susceptibility
Source: BMC Plant Biol. 2019 Dec 6;19:544. doi: 10.1186/s12870-019-2129-8 (PMC6896422; doi:10.1186/s12870-019-2129-8)

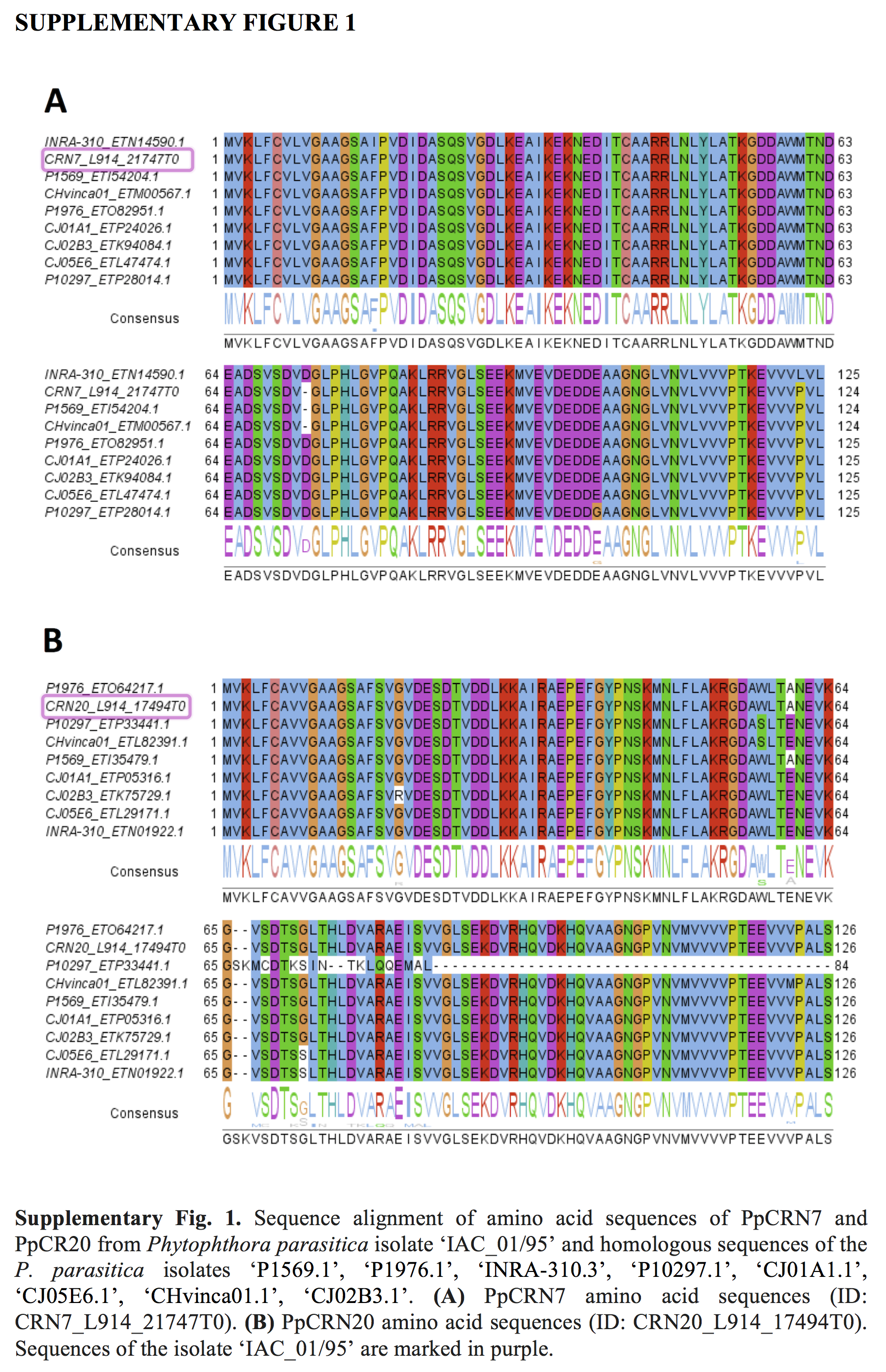

Supplement: Supplementary file 3 — Additional file 3. Fig. 1: Sequence alignment of amino acid sequences of PpCRN7 and ppCRN20 from P. parasitica isolates .TIFF (18,7 MB). [file 12870_2019_2129_MOESM3_ESM.tiff]

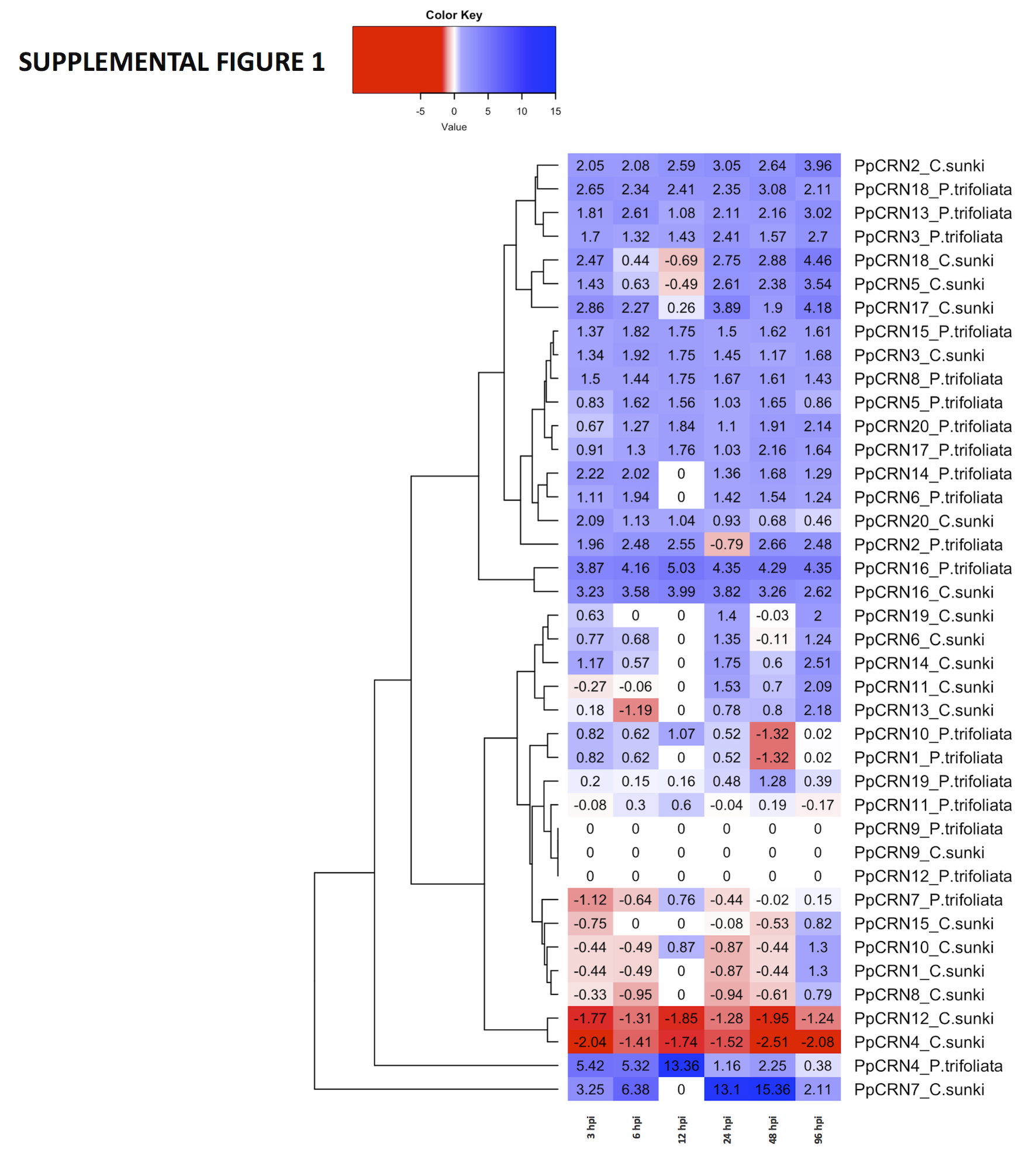

Supplement: Supplementary file 4 — Additional file 4. Fig. 2: PpCRN gene expression analysis during P. parasitica interaction with C. sunki and P. trifoliata .TIFF (20,4 MB). [file 12870_2019_2129_MOESM4_ESM.tiff]

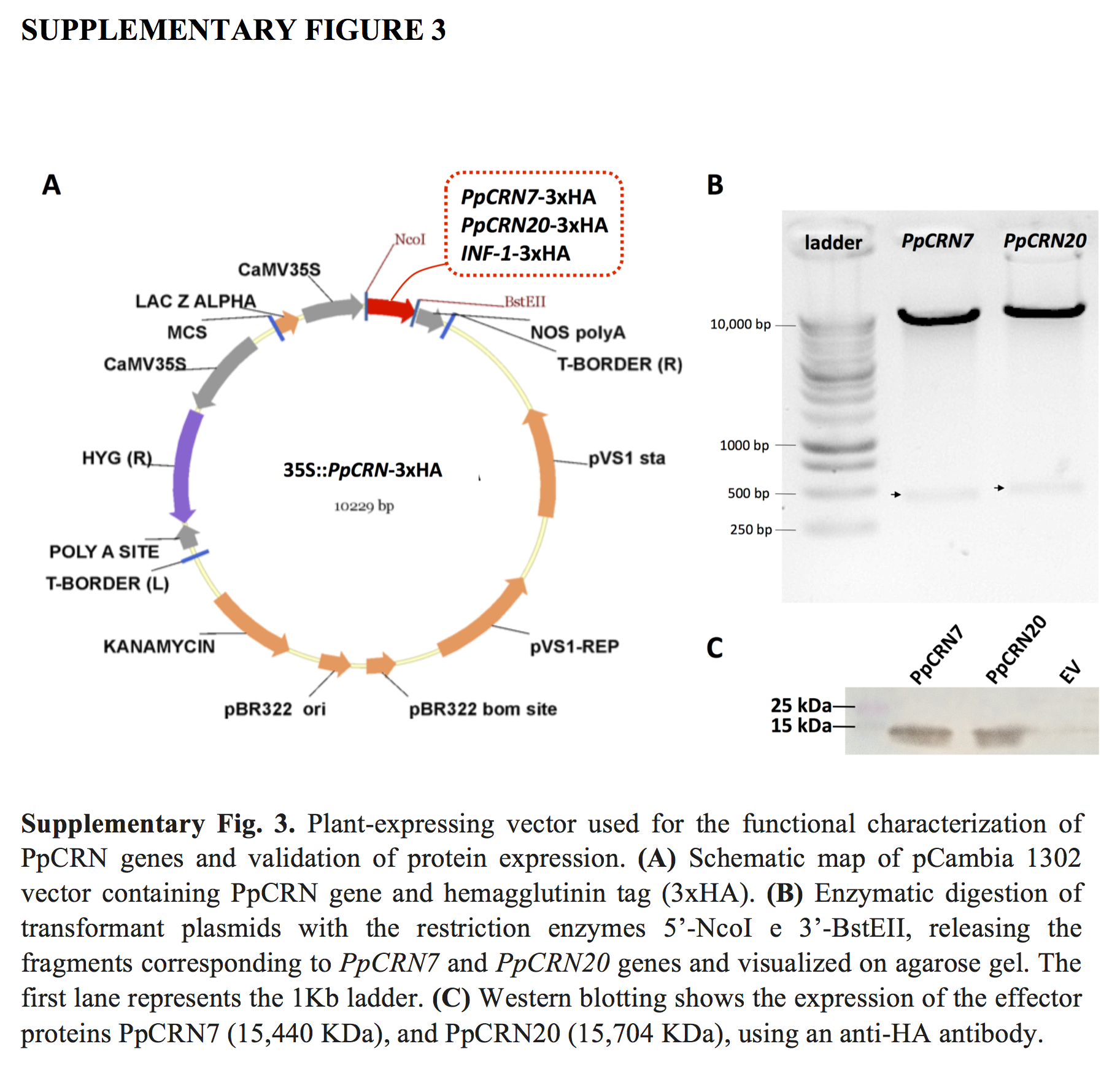

Supplement: Supplementary file 5 — Additional file 5. Fig. 3: Plant-expressing vector used for the functional characterization of PpCRN genes and validation of protein expression .TIFF (12,7 MB). [file 12870_2019_2129_MOESM5_ESM.tiff]

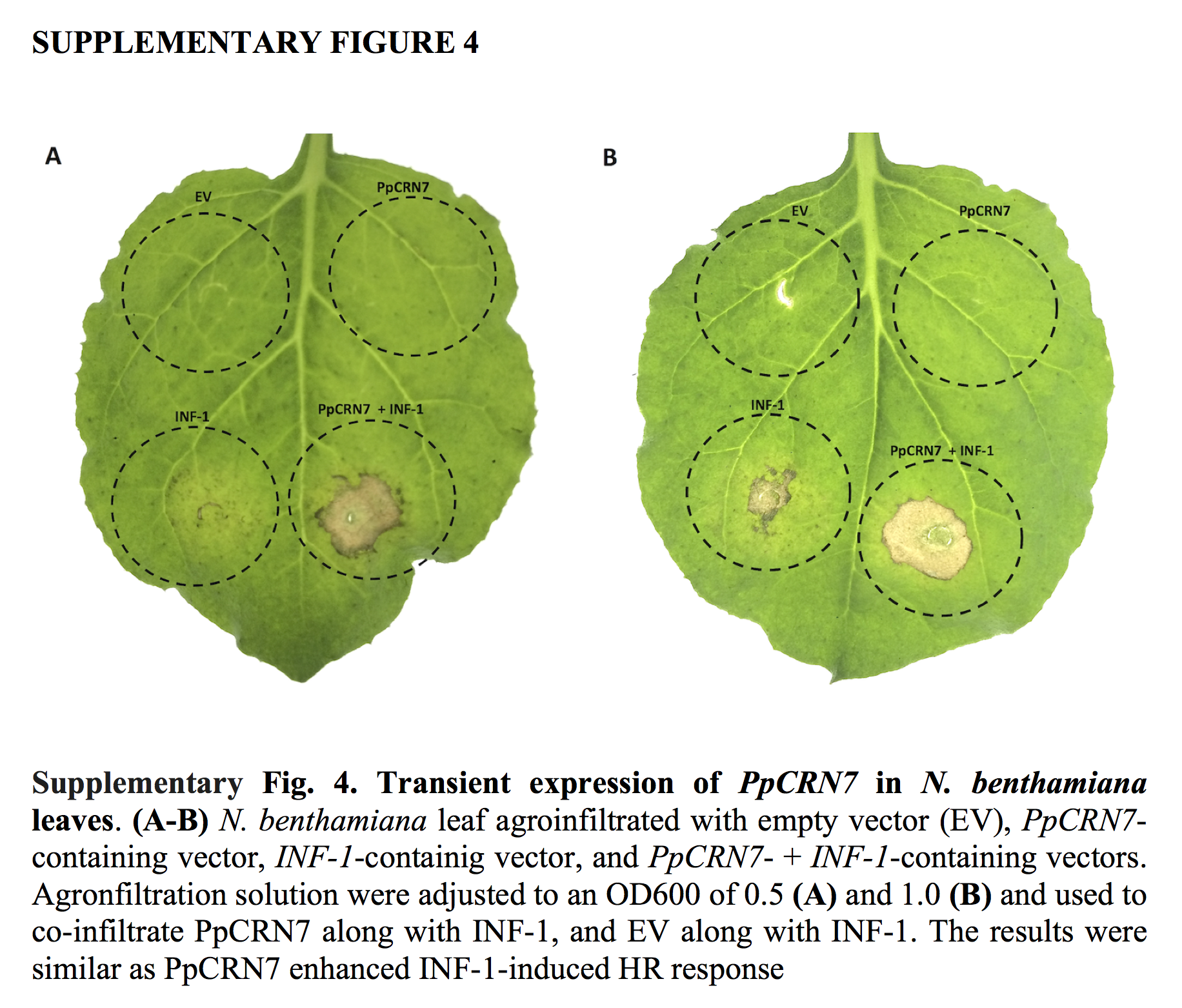

Supplement: Supplementary file 6 — Additional file 6. Fig. 4: Transient expression of PpCRN7 in N. benthamiana leaves .TIFF (11,4 MB). [file 12870_2019_2129_MOESM6_ESM.tiff]
